# Faces with severe front tooth misalignment are particularly perceived: emotional and social perception and eye-tracking

**DOI:** 10.1007/s00056-025-00615-9

**Published:** 2025-10-14

**Authors:** Lisa-Marie Mai, Philipp Meyer-Marcotty, Bernhard Wiechens, Anja Quast, Georg W. Alpers, Antje B. M. Gerdes

**Affiliations:** 1 Department of Orthodontics, University Medical Center Göttingen, Göttingen, Germany; 2 Department of Psychology, School of Social Sciences and Otto Selz Institute, University of Mannheim, Mannheim, Germany

**Keywords:** Orthodontics, Psychology, Malocclusion, Eye movements, Psychosocial aspects, Kieferorthopädie, Psychologie, Malokklusion, Augenbewegungen, Psychosoziale Aspekte

## Abstract

**Objectives:**

Aligned teeth commonly convey a positive impression to other people. This study aimed to examine the effect of malocclusion on adult facial perceptions using self-reported and quantitative measures.

**Materials and methods:**

Facial photographs of 15 adult patients (11 women; 47.73 years, standard deviation [SD] = 8.86; 12 periodontally compromised) with malocclusion were acquired before (MT0) and after treatment (MT1). The control group (CG) comprised 15 age-matched adults (12 women; 42.93 years, SD = 7.50) without malocclusion. In all, 37 laypersons (18 women; 41.06 years) viewed all pictures. Self-report: Participants rated each face regarding dental condition, smile valence, and friendliness. Eye-tracking: Visual attention was analyzed by entry time and fixation time on predefined face areas.

**Results:**

MT0 pictures received significantly (*p* < 0.001) more negative ratings regarding dental condition, smile valence, and friendliness. Ratings for MT1 were comparable to those of the control group. Compared with the CG, MT0 faces showed faster first fixations on the mouth (*p* = 0.005) and had longer fixation durations (36.15%, *p* < 0.001). After treatment, the difference to the CG decreased significantly (12.38%, *p* = 0.014).

**Conclusion:**

Malocclusions have a negative effect on the perception of individuals in terms of gaze behavior and self-reported ratings. These effects decrease after treatment, underlining the effect of orthodontic correction.

**Clinical relevance:**

Orthodontic treatment improves facial perception and the assessment of oral regions, highlighting the psychosocial significance of orthodontic treatment.

## Abstract (de)

**Ziele:**

Eine harmonische Zahnstellung vermittelt häufig einen positiven Eindruck auf andere Menschen. Ziel dieser Studie war es, die Auswirkung von Zahnfehlstellungen auf die Wahrnehmung des Gesichts bei Erwachsenen anhand von Selbstauskünften und quantitativen Messungen zu untersuchen.

**Materialien und Methoden:**

Gesichtsaufnahmen von 15 erwachsenen Patienten (11 Frauen; 47,73 Jahre, Standardabweichung [SD] = 8,86; 12 parodontal erkrankt) mit Malokklusion wurden vor (MT0) und nach der Behandlung (MT1) aufgenommen. Die Kontrollgruppe (CG) umfasste 15 altersentsprechende Erwachsene (12 Frauen; 42,93 Jahre, SD = 7,50) ohne Malokklusion. Insgesamt sahen sich 37 Laien (18 Frauen; 41,06 Jahre) alle Bilder an. Selbstauskunft: Die Teilnehmenden bewerteten jedes Gesicht hinsichtlich des Zahnzustands, der Valenz des Lächelns und der Freundlichkeit. Eye-Tracking: Die visuelle Aufmerksamkeit wurde anhand von Eintritt der ersten Fixierung und Dauer der Fixierung auf vorher festgelegten Bereichen des Gesichts analysiert.

**Ergebnisse:**

MT0-Bilder erhielten signifikant (*p* < 0,001) negativere Bewertungen hinsichtlich Zahnzustand, Valenz des Lächelns und Freundlichkeit. Die Bewertungen für MT1-Bilder waren vergleichbar mit denen der Kontrollgruppe. Im Vergleich zur CG zeigte sich bei MT0-Gesichtern eine schnellere erste Fixierung auf den Mund (*p* = 0,005) und eine längere Fixierungsdauer (36,15%, *p* < 0,001). Nach der Behandlung verringerte sich der Unterschied zur CG signifikant (12,38%, *p* = 0,014).

**Schlussfolgerung:**

Zahnfehlstellungen wirken sich negativ auf die Wahrnehmung des Einzelnen aus, was Blickverhalten und Selbsteinschätzungen betrifft. Diese Effekte verringern sich nach der Behandlung und verdeutlichen den positiven Einfluss der kieferorthopädischen Therapie.

**Klinische Relevanz:**

Eine kieferorthopädische Behandlung verbessert die Wahrnehmung des Gesichts und die Bewertung der Mundregion. Dies unterstreicht die psychosoziale Bedeutung einer kieferorthopädischen Behandlung.

## Introduction

The focus on orthodontic treatment in adults has steadily increased over the past 20 years. Orthodontic treatment aims to improve both function and aesthetics of the dentition. The severity of malocclusion and the need for treatment are commonly assessed using standardized indices. Among these, the Dental Aesthetic Index (DAI) [10] and Little’s Irregularity Index (Little) [14] are widely accepted tools which quantify aesthetic impairment and dental irregularities and help to objectively evaluate treatment necessity.

The interdisciplinary orthodontic/periodontic treatment in adult patients has also seen a steady increase, with the primary motivation of patients to retain their oral health and improve the appearance of their teeth [22]. Dental malocclusions can, among many other causes, also be due to periodontitis, with characteristic proclinations, irregular spacing, rotations and over-eruptions in the upper front tooth segment [8].

According to the recent S3 guideline by the European Federation of Periodontology (EFP), an interdisciplinary treatment strategy is essential to stabilize the functional and aesthetic condition of the teeth and improve the quality of life in patients with stage IV periodontitis [7]. The implementation of the orthodontic treatment sequence supports the possibility of long-term periodontal care, such as plaque control, aesthetics, and dental function [21].

In Germany, according to the Fifth German Oral Health Study (DMS V) by the Institute of German Dentists, every second (52%) younger adult aged 35–44 years was affected by periodontal disease in 2016. Among seniors aged 65–74 years, 65% had moderate to severe periodontal disease [3]. Over 50% of patients with periodontitis exhibit unphysiological tooth migration with the aspects described above [2]. This illustrates that the clinical picture of periodontitis must be considered in orthodontic planning and treatment. In a recently published study—conducted by the Department of Orthodontics at the University Medical Center Göttingen—the association between moderate to severe bone loss due to periodontitis and severe pathological tooth migration was shown [20].

Whether and how malocclusions affect the visual face perception has not yet been investigated. The method of noninvasive eye-tracking has been successfully used in numerous studies on the visual perception of orthodontic patients [18, 19, 23]. With this technique, the eye movements of the subjects are recorded using a small camera while looking at pictures of patients on a screen, thereby mapping visual attention processes. The recording of the combination of saccades and fixations makes it possible to systematically analyze dwell time and frequency on specific areas of the picture that are of particular interest, known as areas of interest (AOIs).

With this study it should be investigated how orthodontic treatment contributes to improving the self-reported assessment of patients regarding the psychosocial impact by others. As null hypothesis it was assumed that the test raters do not rate faces with or without severe front tooth misalignment differently. In this way, it should also be clarified whether it is possible to reduce psychological distress and improve the appearance of patients through orthodontic therapy.

## Participants and methods

The present study was performed by the Department of Orthodontics at the University Medical Center Göttingen in collaboration with the Department of Psychology, School of Social Sciences and Otto Selz Institute of the University of Mannheim. The Ethics Committee of the University Medical Center Göttingen gave approval for this study (ethics reference number: 18/6/22).

### Orthodontic indices

The DAI combines the aesthetic aspect with clinical need into a single score by evaluating and weighting ten different parameters, with a score of ≥ 31 indicating the need for orthodontic treatment [10].

The Little measures the linear distances between anatomical contact points from canine to canine. Originally, this index was developed to assess anterior crowding only in the lower jaw [14]. For this study, it was also applied to the upper jaw, and the average of the upper and lower jaw values was used to determine the severity of irregularity.

### Portrayed individuals

For the test pictures, 15 patients were selected. The patients had to be at least 30 years old; in addition, initial and final models with a DAI ≥ 31 and Little ≥ 4 as well as a smiling picture before and after orthodontic therapy were available. Furthermore, the 15 individuals in the control group (CG) had to be consistently below these orthodontic values. All portrayed individuals were required to have a healthy overall condition of their anterior teeth. Individuals with a congenital malformation (cleft lip and palate/craniofacial syndrome), current orthodontic treatment, insufficient remaining teeth for index collection or missing front teeth were excluded from the study.

### Picture categories

**Fig. 1:**
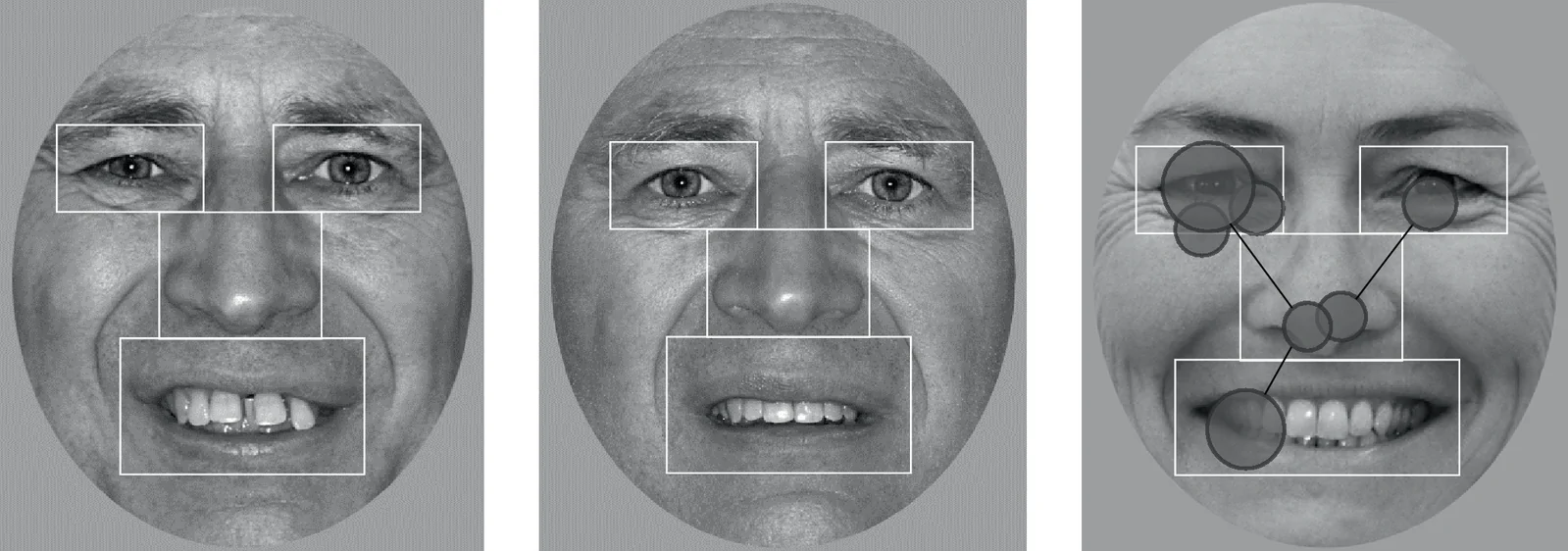
Standardized example pictures of the three groups MT0 (preorthodontic; left), MT1 (postorthodontic; middle), and CG (control group without orthodontics; right). The white boxes represent the areas of interest (AOIs) “eyes”, “nose”, and “mouth”. On the right, saccades and fixations recorded by the eye-tracker are shown as an example; circles indicate fixations, with larger circles representing longer fixation durations Standardisierte Beispielbilder der drei Gruppen MT0 (vor der kieferorthopädischen Behandlung; links), MT1 (nach der kieferorthopädischen Behandlung; Mitte) und CG (Kontrollgruppe ohne kieferorthopädische Behandlung; rechts). Die weißen Kästen stellen die „areas of interest“ (AOIs) dar: Augen, Nase, Mund. Rechts exemplarisch vom Eye-Tracker aufgezeichnete Sakkaden und Fixationen: Kreise zeigen Fixationen an, größere Kreise stehen für längere Fixationszeiten

Frontal photographs were used as visual stimuli and presented to the participants. The pictures were taken with a digital single-lens reflex camera (Canon EOS 50D from Canon, Tokyo, Japan) and digitally edited with image editing software (Adobe Photoshop from Adobe Systems, San Jose, CA, USA) for the study to conceal visual distractions and color the pictures in grayscale. The pictures were also cropped to uniform ovals and placed on a standardized gray background of 660 × 800 pixels to minimize perceptual variability, such as colors and backgrounds, and thus their influence on image processing. Overall, the portrayed individuals were divided into following groups (Fig. 1):

- MT0: Patients with malocclusion (preorthodontic)
- MT1: Patients at the time of successful correction of the malocclusion (postorthodontic)
- CG: Control group without malocclusion (without orthodontics)

Each of these groups consisted of 15 individuals (MT0/MT1: 11 females; CG: 12 females). The individuals in group MT0 and MT1 were the same patients, photographed before and after orthodontic treatment. Twelve out of 15 individuals in the MT0/MT1 group were periodontally compromised, whereby all individuals in the CG were periodontally healthy. The pictures of the CG were mirrored to counteract bias regarding the number of pictures per group, resulting in a total of 60 pictures per session. The average age of the MT0 and MT1 group was 47.73 years (minimum [Min] = 30, maximum [Max] = 59, standard deviation [SD] = 8.86), and that of the CG was 42.93 years (Min = 31, Max = 54, SD = 7.50).

### Trial design: rating task and eye-tracking task

The two tailed experiment was conducted in a separate room at the Department of Orthodontics at the University Medical Center Göttingen where the room lighting was consistent across all sessions. Data from 37 participants (18 females; 41.06 years) for the questionnaire-based rating task and eye-tracking task were included in this study. All participants were laypeople with no medical background. Overall, including instructions, the rating task and the eye-tracking task, the study took an average of 45 min per participant. Participation was voluntary and no financial compensation was provided. The experimental design was developed in accordance with established protocols from previous studies [6, 23].

#### Rating task

The questionnaire included 45 images, each accompanied by four questions in which participants subjectively rated the previously viewed images, excluding the mirrored control group photos. Ratings were provided on Likert scales ranging from 0 to 10 (0 = very poor, 10 = very good) across four dimensions. For reasons of space, only three dimensions are presented below: dental condition, valence, and friendliness.

#### Eye-tracking task

Instructions, test runs and stimuli were presented to the participants using a presentation software (Presentation, Version 17.2, Neurobehavioral Systems Inc., Berkley, CA, USA) at a resolution of 1680 × 1050 pixels on a 22-inch LCD monitor (DELL Technologies Inc., Round Rock, TX, USA), with participants seated approximately 60 cm away on a nonmovable chair. Eye movements were recorded at 250 Hz by an eye-tracker (SMI Remote 250 from SensoMotoric Instruments, Teltow, Germany) mounted as a narrow bar under the monitor and measured by a video-supported infrared camera (gaze position accuracy < 0.4°, spatial resolution < 0.1°, temporal resolution 60 Hz). Before each trial, the system was individually calibrated for each participant to ensure accurate measurements by using a 11-point-calibration method. For the evaluation of the eye-tracking data, fixations were defined as those data points of the gaze path that were limited to a maximum viewing angle radius of 2.02° for at least 80 ms.

After successful calibration, participants were shown pictures of smiling individuals from picture category MT0, MT1, and CG, with each stimulus presented for 5000 ms after the participant fixated on a cross in the center of the screen for 1000 ms. The pictures were shown in randomized order and randomly on the left or right side of the screen to trigger an initial saccade from the fixation cross in the center of the screen towards the picture and to prevent bias in the viewing path.

#### Eye-tracking parameters

The three most significant AOIs were defined as “eyes”, “nose” and “mouth”. The following two parameters were collected in relation to the eye-tracking experiment: 1) The “entry time” describes the time in milliseconds until an AOI is first fixated. 2) The “fixation time” is the cumulative total duration of fixations in an AOI, serving as a basis for later attention processes [1].

### Statistics

For the evaluation of the data recorded by the eye-tracker, the software BeGaze (version 3.7 from SensoMotoric Instruments, Teltow, Germany) was used.

Subsequently, the eye-tracking/questionnaire data were analyzed descriptively and by a multifactorial analysis of variance (ANOVA) with repeated measures using the statistical program SPSS Statistics (version 29.0.1.0, IBM Corporation, Armonk, NY, USA), with the significance level set at *p* = 0.05. Sphericity was checked using Mauchly’s test. Since there was a violation of the sphericity assumption regarding the picture categories both independently and dependent on the AOIs, a Greenhouse–Geisser correction of the degrees of freedom was applied (*p* = 0.05). Finally, Bonferroni-corrected post hoc tests were conducted.

## Results

### Rating parameter: dental condition

**Fig. 2:**
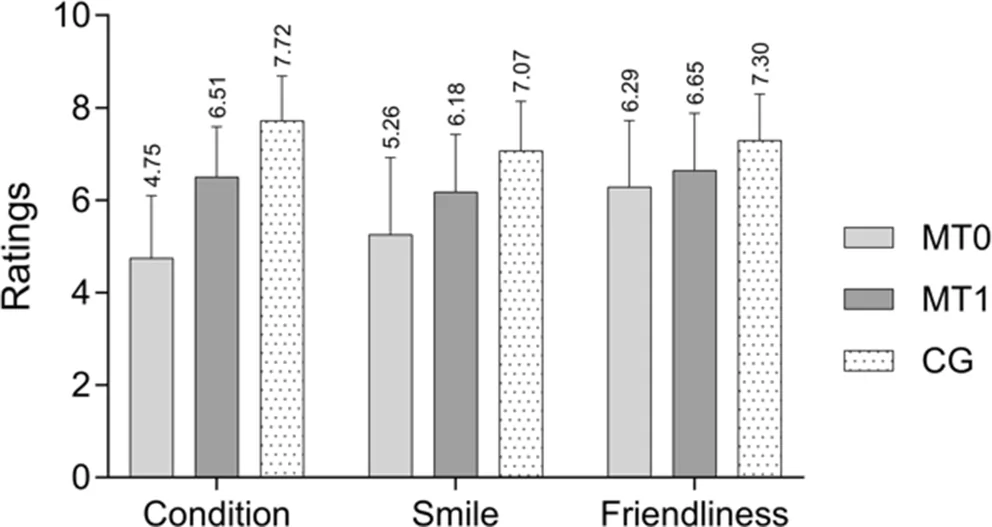
Representation of the ratings for the groups MT0 (preorthodontic), MT1 (postorthodontic), and the control group (CG). The ratings assessed the dental condition (condition), the smile, and the friendliness of the portrayed individual (smile, friendliness). The values provided include mean and standard deviation Darstellung der Bewertungen für die Gruppen MT0 (vor der kieferorthopädischen Behandlung), MT1 (nach der kieferorthopädischen Behandlung) und Kontrollgruppe (CG). Bewertet wurden der Zahnzustand („condition“), das Lächeln und die Freundlichkeit („smile“, „friendliness“) der betrachteten Person. Die angegebenen Werte umfassen Mittelwerte und Standardabweichungen

There was a significant effect related to the parameter dental condition for the individuals presented in the pictures, F (1.29, 41.34) = 171.31, *p* < 0.001. The Bonferroni-corrected post hoc tests showed significantly (*p* < 0.001) lower ratings for pictures from category MT0 than for those from category MT1 and from the CG (MDiff = −1.79, 95% CI [−2.17, −1.41] and MDiff = −3.03, 95% CI [−3.57, −2.48]). The ratings for pictures from image category MT1 were also significantly (*p* < 0.001) lower than the ratings for the pictures from the CG (MDiff = −1.24, 95% CI [−1.52, −0.96]), with the portrayed individuals from picture category MT0 being rated 27.04% lower than those from MT1 and 38.47% lower than those from CG (Fig. 2).

### Rating parameter: valence and friendliness

The valence of the smile (pleasant vs. unpleasant) was rated significantly differently across the three picture categories, F (1.28, 41.10) = 61.27; *p* < 0.001, with the Bonferroni-corrected post hoc tests showing significantly lower ratings for both picture categories MT0 and MT1 compared to CG (MT0 vs. CG: MDiff = −1.84, 95% CI [−2.38, −1.29]; MT1 vs. CG: MDiff = −0.87, 95% CI [−1.13, −0.61]) as well as for picture category MT0 compared to MT1 (MDiff = −0.97, 95% CI [−1.37, −0.56]). The mean of the smile ratings of the patients in the CG was 34.41% higher than the ratings for category MT0 and 14.40% higher than for category MT1 (Fig. 2).

Significant differences were also observed in the assessment of the parameter expression of friendliness, F (1.38, 44.24) = 32.99, *p* < 0.001, between the picture categories MT0, MT1 and CG. The Bonferroni-corrected post hoc tests revealed significantly (*p* < 0.001) lower ratings for MT0 and MT1 compared to CG (MDiff = −1.03, 95% CI [−1.45, −0.61] and MDiff = −0.66, 95% CI [−0.91, −0.41]), as well as between MT0 and MT1 (MDiff = −0.37, 95% CI [−0.65, −0.09]), with MT0 having the lowest mean, which was 5.41% lower than that for MT1 and 13.84% lower than that for CG (Fig. 2).

### Eye-tracking: entry time

For the eye-tracking parameters, two separate repeated measure analysis of variance (ANOVAs) were conducted with the within-subject factors picture category (MT0, MT1, CG) and AOI (“eyes”, “nose”, “mouth”).

The ANOVA revealed a significant interaction of the parameters AOI and picture category, F (2.78, 91.60) = 4.60, *p* = 0.006, showing a significantly (*p* = 0.005) shorter entry time for pictures in category MT0 compared to pictures in the CG (MDiff = −130.56, 95% CI [−226.62, −34.51]) in the Bonferroni-corrected post hoc tests.

When considering the entry time across the different picture categories independent of the AOIs, no significant main effects were found, F (1.40, 46.34) = 3.26, *p* = 0.064, nor when considering different AOIs independent of the picture category, F (2.00, 66.00) = 2.75, *p* = 0.071 (Table 1).

**Table 1:** Different entry times^a^ regarding areas of interest (AOIs) Unterschiedliche Eintrittszeiten^a^ in die „areas of interest“ (AOIs)

	Eyes	Nose	Mouth
*MT0*			
M [ms]	1514.40	1825.44	1535.71
SD [ms]	474.40	857.17	711.80
Min [ms]	175.10	570.56	669.46
Max [ms]	2477.66	4927.70	3921.60
*MT1*			
M [ms]	1484.77	1735.40	1710.50
SD [ms]	467.80	666.63	890.19
Min [ms]	721.76	610.03	595.31
Max [ms]	2809.36	3217.23	5116.50
*CG*			
M [ms]	1477.36	1934.60	1754.09
SD [ms]	499.80	618.95	696.38
Min [ms]	738.44	871.18	518.54
Max [ms]	3142.38	3096.58	3565.80

*Min* Minimum, *Max* Maximum, *M* Mean, *SD* standard deviation, *MTO* preorthodontic, *MT1* postorthodontic, *CG* control group

^a^The entry time refers to the measurement of the time until an AOI is first fixated [ms]

### Eye-tracking: fixation time

The ANOVA revealed a significant interaction of AOI and picture category, F (1.97, 70.77) = 17.94, *p* < 0.001. The Bonferroni-corrected post hoc tests showed a significantly (*p* < 0.001) higher fixation time for picture category MT0 than MT1 (MDiff = 48.32, 95% CI [21.52, 75.11]) as well as for CG (MDiff = 79.10, 95% CI [47.74, 110.46]).

Furthermore, a significant main effect of picture category, F (2.00, 72.00) = 25.54, *p* < 0.001, was revealed, showing a significantly (*p* = 0.014) higher fixation time in picture category MT1 than in CG (MDiff = 30.78, 95% CI [5.21, 56.35]).

In addition, a significant main effect of the parameter AOI, F (1.39, 50.13) = 16.56, *p* < 0.001, was revealed, showing a significantly (*p* < 0.001) higher fixation time for the AOI “eyes” as well as the AOI “mouth” compared to the AOI “nose” (MDiff = 371.76, 95% CI [194.00, 549.53] and MDiff = 678.98, 95% CI [356.19, 1001.77]; Table 2).

**Table 2:** Different fixation times^a^ regarding areas of interest (AOIs) Unterschiedliche Fixierungszeiten^a^ in den „areas of interest“ (AOIs)

	Eyes	Nose	Mouth
*MT0*			
M [ms]	700.28	344.18	1220.07
SD [ms]	393.10	264.45	787.25
Min [ms]	15.87	0.00	10.40
Max [ms]	1632.98	1353.42	3343.25
*MT1*			
M [ms]	735.45	377.02	1007.11
SD [ms]	413.44	323.53	709.29
Min [ms]	14.67	0.00	0.00
Max [ms]	1834.21	1630.19	2936.60
*CG*			
M [ms]	765.93	365.17	896.14
SD [ms]	382.12	320.68	696.17
Min [ms]	39.61	15.73	0.00
Max [ms]	1572.51	1671.24	3215.82

*Min* Minimum, *Max* Maximum, *M* Mean, *SD* standard deviation, *MTO* preorthodontic, *MT1* postorthodontic, *CG* control group

^a^Mean viewing duration of the individual AOIs [ms]

## Discussion

This study aimed to investigate how dental malocclusion affects facial perception and social evaluation. Both self-reported ratings and quantitative eye-tracking data provide insights into the psychosocial relevance of dental malocclusion.

### Rating parameters

The rating of the pictures by the participants revealed the following pattern: The condition of the teeth, the valence of the smile and the expression of friendliness of the patients were rated the lowest in patients with malocclusion before therapy, followed by patients after therapy. The control individuals without dental malocclusion were rated the highest in both categories.

Despite all groups having healthy anterior teeth, the overall dental condition was rated significantly worse in the patients with a malocclusion. It is worth investigating in future studies whether dental malocclusion alone leads to other negative evaluations, for example, if the oral hygiene or oral health of the affected person is rated as less healthy.

It could also be examined whether there is a difference in the evaluation of the dental condition of patients with dental malocclusion between participants who themselves exhibit dental malocclusion or not (cf. [9]), as people with dental malocclusion are generally less satisfied with their oral health than patients without malocclusion [11] which might cause them to evaluate others differently.

The smile is a centrally important feature for facial attractiveness and the display of emotions, strongly influenced by malocclusion [26]. The results thus support the assumption that people with attractive appearance are perceived as friendlier, which is also reinforced by numerous studies showing that these people are perceived as more sociable and socially competent [4, 5, 13, 27, 28].

In addition, it can be assumed that the smile of patients with dental malocclusion was rated as less friendly because these patients, due to their malocclusion, especially those with anterior tooth migrations, have significantly reduced self-esteem [12] and tend to show a less natural and more forced smile, as insecurity and tension affect their facial expression. This assumption is supported by a study, which found that visible malocclusion and dental anomalies (e.g., caries, missing teeth, unaesthetic restorations, or discolorations) cause affected patients to try to cover these less aesthetic areas with their lips out of embarrassment, resulting in less frequent smiling overall [16].

Interestingly, the ratings for the orthodontically treated patients did not reach the levels of those for the control group. This suggests that while orthodontic treatment improves the appearance, it may not fully restore social perception to the level of individuals without a history of malocclusion. Facial features shaped during development, such as subtle asymmetries or skeletal structure, may remain unchanged and continue to influence observers’ impressions, which highlights the complexity of facial perception. Furthermore, periodontal bone loss could affect the appearance of the smile, which typically may not be reversed by orthodontic treatment.

### Eye-tracking

The entry time describes the duration until an AOI is first fixated and showed the shortest time for the AOI “eyes” in all three picture categories, followed by the AOI “mouth”. Among the patients with malocclusion before therapy, the mouth region was fixated the quickest, followed by the patients after therapy. Notably, the reduced entry time to the mouth region of the patients with malocclusion before therapy was at the expense of the entry time to the eyes, as the entry time to the AOI “eyes” in this group was longer than in the other two groups. It can thus be concluded that people with anterior dental malocclusion are looked at the mouth more quickly. This aspect, involving a change in the facial perception registered through the quantitative method of eye-tracking, includes the risk of stigmatization due to the malocclusion.

The mean viewing duration of the AOIs was measured using the fixation time. This parameter serves as a basis for subsequent attentional processes as it measures sustained attention [1]. Here, the AOI “mouth” showed the longest fixation time in all picture categories, followed by the AOI “nose” and, lastly, the AOI “eyes”. Overall, the longest sustained attention was on the mouth region of patients with malocclusion before therapy.

Regarding smile, the most important means of communication [17], dental alignment has the greatest psychosocial impact [15]. The regions of the eyes and mouth attract central attention in social interactions [25], which aligns with the results of this study.

All in all, the eye-tracking data showed a different visual perception in the initial and sustained attention between the images of the group MT0, MT1 and CG. Therefore, it can be hypothesized that malocclusion has a direct negative effect on human interaction.

Further research should also address the observers’ visual response to smiles with or without malocclusion since unobtrusive technology is now available to measure mimicry (cf. [24]).

### Limitations

To maximize validity, we used a convenience sample of pictures which may not have been perfectly balanced in terms of facial features aside from the malocclusion. Importantly, however, as perception of pictures was comparable after the intervention, we conclude that patient and control participants did not grossly differ on relevant features except for the malocclusion.

Moreover, to keep the number of pictures and trials small, we only presented pictures with smiles. The finding that the perception of positive expressions is impacted by malocclusion may stimulate future studies to compare a wider range of facial expressions. This is important as the impact may be even more pronounced in facial expressions with negative valence.

Furthermore, it must be noted that the treatment and control groups differed in terms of periodontal status. While most but not all patients in the treatment group were periodontally compromised, the control group consisted of periodontally healthy individuals only. Future studies should further investigate how perception differs between patients with severe malocclusion with or without periodontal involvement to better isolate the role of periodontal disease in social perception.

The pictures of the patients were previously edited in Adobe Photoshop to conceal visual distractions, color the photos in grayscale, crop them to uniform ovals and place them on a standardized background to minimize perceptual variability between the pictures. This allowed for clear and comparable results to be generated. However, the participants might have viewed the patients differently than in everyday encounters.

Furthermore, it cannot be ruled out that facial features outside the analyzed regions such as wrinkles or subtle mimic-related features may have influenced the perception. This aspect should be addressed in future studies aiming for a more comprehensive assessment of facial affects.

This study was based on an established setup [18, 19] and the eye movements were also recorded at 60 Hz, which already allowed a significant difference in the three picture categories mentioned above to be determined. In addition, a 238 Hz camera could have been used to better represent initial and subconscious eye movements (cf. [8]), as the initial stimulus response occurs already within 300 ms [24].

## Conclusion

It was shown that pathological tooth migration due to periodontal disease significantly influenced the social visual attention and evaluation:

- The mouth region of patients with pathological tooth migration received more attention and was rated more negatively by laypersons, suggesting possible stigmatization due to dental malocclusion.
- Orthodontic treatment significantly improved the facial perception and evaluation of the mouth region of patients with pathological tooth migration.
- In cases where corrective treatment is not possible, available or not preferred by the patient, psychosocial support may be offered to reduce the effects of potential stigmatization. While substantial malocclusion is an indication for correction, not all aesthetic advantages may necessitate intervention.

These results highlight the psychosocial impacts of dental malocclusion and motivate further research regarding the patient’s own perspective.

## Data Availability

The data supporting the findings of this study are not publicly available. The datasets are securely stored on a password-protected institutional computer at the Department of Orthodontics, University Medical Center Göttingen. Access to the data can be granted upon reasonable request and with permission from the corresponding author and the institution.
